# Experiments on Nonlocal Fluid‐Flow Metamaterials

**DOI:** 10.1002/advs.77912

**Published:** 2026-09-27

**Authors:** Ke Wang, Yi Chen, Martin Wegener

**Affiliations:** ^1^ Institute of Nanotechnology Karlsruhe Institute of Technology (KIT) Karlsruhe Germany; ^2^ Institute of Applied Physics Karlsruhe Institute of Technology (KIT) Karlsruhe Germany; ^3^ Marine Science and Technology Domain Beijing Institute of Technology (BIT) Zhuhai China

**Keywords:** backward flow, flow control, fluid‐flow metamaterial, nonlocal interaction, streamlines

## Abstract

Metamaterials for controlling fluid flow have recently attracted considerable attention as an emerging new direction in the field of metamaterials. Herein, nonlocal metamaterials composed of one‐dimensionally periodic networks of standard microfluidic tubes and connectors are designed, comprising nearest‐neighbor (local) and beyond‐nearest‐neighbor (nonlocal) connections of order *N* = 2,3,4. The relative importance of laminar water flow through the nonlocal and local tubes is controlled by introducing tailored constrictions into the local tubes that serve as water‐flow resistors. Unusual alternating backward/forward laminar flows of water that depend on the metamaterial parameters and on the boundary conditions are observed. Qualitatively, the backward/forward flows can be seen with the naked human eye by following microparticles introduced into the water. Quantitatively, the backward/forward flows are characterized and mapped by digital image analysis of movies taken with a camera. The experimental findings agree well with finite‐element calculations of the network's fluid streamlines that exhibit loop‐like behavior as well with calculations of the evanescent zero‐frequency Bloch eigenmodes of a simplified discrete periodic model, providing an intuitive understanding.

## Graphical abstract

A nonlocal fluid‐flow metamaterial composed of standard microfluidic tubes and connectors is designed. Hence, the current of the system at one position depends on the gradient of fluid flow at many other positions, leading to unusual alternating backward and forward laminar flows of water. This work offers new insights and design strategies for controlling fluid flows by metamaterials.

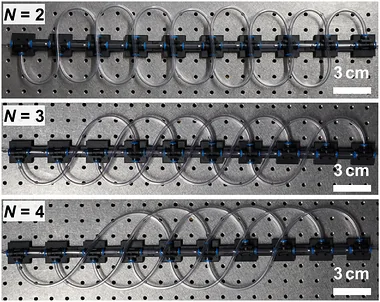

## 1 Introduction

Spatially nonlocal interactions are ubiquitous in crystals and materials. For example, in NaCl crystals, the Coulomb forces between positively charged sodium and negatively charged chlorine ions lead to an oscillatory long‐range Coulomb interaction way beyond nearest‐neighbor interactions that is summarized by the Madelung constant [1]. Brillouin already studied the effect of these beyond‐nearest‐neighbor interactions on the crystal's phonon dispersion relation [2]. The Ruderman‐Kittel‐Kasuya‐Yosida (RKKY) interaction couples localized spins in metals or semiconductors over distances far beyond the nearest neighbors [3]. Long‐range coupling of excitons in semiconductors can strongly modify their dispersion relation [4].

While the strengths of such beyond‐nearest‐neighbor interactions are determined by nature in ordinary materials, they can be nearly freely designed in metamaterials [5, 6, 7, 8, 9, 10, 11, 12, 13, 14]. Here we define metamaterials [5] as designed (by human or artificial intelligence) composites with effective properties that go beyond the properties of the ingredients. The fluid‐flow networks discussed in this paper as well as many other related networks [9, 15, 16, 17, 18, 19, 20, 21, 22, 23, 24, 25, 26, 27, 28] follow this definition. They are composed of man‐made building blocks or unit cells and man‐made interactions [9, 15, 16, 17, 18, 19, 20, 21, 22, 23, 24, 25, 26, 27, 28]. For example, one can design lattices in which exclusively nearest‐neighbor interactions (*N* = 1) plus next‐to‐nearest neighbor interactions (*N* = 2) occur. The resulting effective material behavior has been studied for elastic waves [9, 18, 19, 20], acoustic waves [21, 22, 23], static elasticity [24], radio‐frequency waves [25, 26, 27], and static electric conduction [28]. In all of these systems, there is no threshold for nonlocality with increasing strength of the beyond‐nearest‐neighbor interactions. However, it only makes sense to refer to (meta)materials as being ‘nonlocal’ if some observable shows marked deviations from ordinary local behavior. On an effective‐medium level, local behavior means that some current (e.g., the momentum current, charge current, or mass current) at position *x* is proportional to the gradient of some potential at the same position *x*. For nonlocal behavior, the same current depends on the gradient at other positions $x^{\prime }\neq x$ as well. For periodic structures, the microscopic origin of this macroscopic effective behavior can be the mentioned beyond‐nearest‐neighbor interactions between different sites of the periodic lattice. This definition for nonlocal metamaterials has been discussed in more detail in ref. [11].

A common scheme in such nonlocal metamaterials [11] has been the occurrence of backward waves or backward flows, respectively. These contributions move opposite to the intuitively expected direction or opposite to the main wave or flow direction. If the backward‐wave contributions are larger than the forward‐wave contributions, the behavior can be mapped onto an effectively negative refractive index [29, 30]. However, backward flows or waves can also exist for positive refractive indices [31, 32]. The interplay between forward and backward flows has not been observed directly in experiments so far. In stationary situations, alternating backward/forward flows result from spatially oscillating and slowly exponentially decaying evanescent Bloch modes of the periodic system at zero frequency [24].

Here, we experimentally and theoretically study the effects of nonlocal interactions for one‐dimensionally periodic fluid‐flow metamaterials. The combination of metamaterials and fluid flows has recently attracted attention as an open frontier [33, 34, 35, 36, 37], as summarized in a multi‐author perspectives article [38]. Herein, we observe these backward flows directly by zooming into individual unit cells of the metamaterial and by detecting the motion of microparticles in the water. The backward flows can strongly influence the overall viscous‐fluid current through the network as a function of its overall length. Counter‐intuitively, for fixed pressure difference between the two ends of the 1D network, the fluid current can increase when increasing the length of the nonlocal network.

At first sight, our fluid‐flow lattices may appear somewhat similar to the known Tesla valve [39, 40, 41]. However, there are more dissimilarities than similarities: Physically, the Tesla valve exhibits asymmetric flow resistance for left and right inflow condition, while our architectures are symmetric. The Tesla valve serves as an asymmetric element in the regime of nonlinear turbulent fluid flow, we discuss laminar flow. Geometrically, the periodic lattice of the Tesla valve has only nearest‐neighbor connections, in our work beyond‐nearest‐neighbor connections are the key. The backward flows in the Tesla valve mainly occurs in the side branches and they originate from tubes that are geometrically bent backwards. In our lattices, the backward flows do not result from backward‐bent tubes, but result from the nature of the spatially oscillating static Bloch eigenfunctions of the nonlocal network.

## 2 Results

### 2.1 Experiments

**FIGURE 1:**
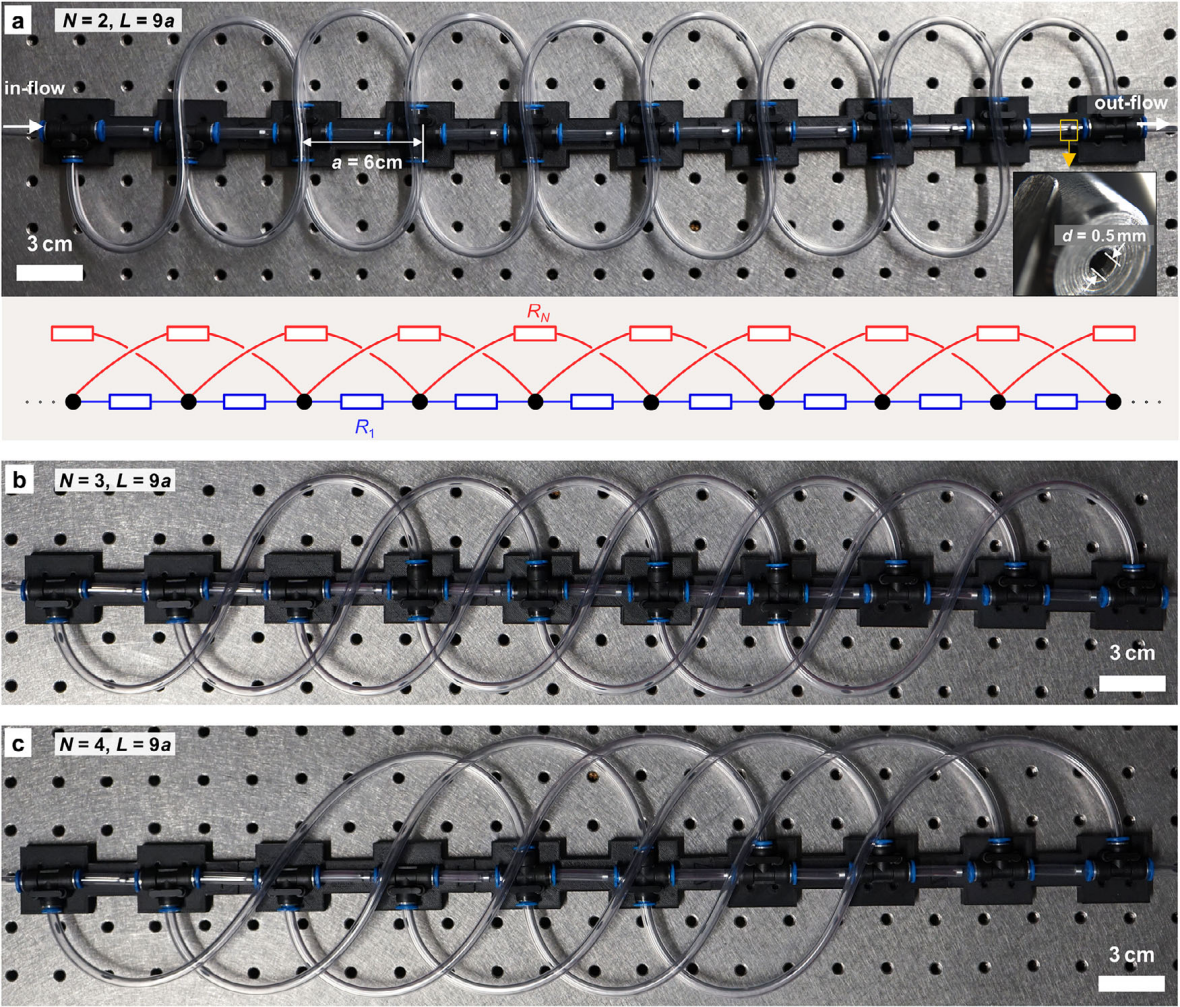
Nonlocal fluid‐flow in a 1D periodic microfluidic metamaterial. (a) The upper part is a photograph of a 1D metamaterial with period *a*  =  6 cm. Straight tubes and S‐shaped tubes provide the nearest‐neighbor (local) and second‐nearest‐neighbor (nonlocal, *N*  =  2) connections, respectively. Hollow aluminum cylinders are used as constrictions to tailor the flow resistance of the local tubes (see inset figure on the right). The lower part is the discrete equivalent fluid‐flow circuit model. Herein, *R*_1_ represents the resistance of nearest‐neighbor (local) connections, and *R_N_* represents the resistance of second‐nearest‐neighbor (nonlocal) connections. (b) Photograph of nonlocal fluid‐flow metamaterial with nonlocal order *N*  =  3. (c) Same as (b), but with nonlocal order *N*  =  4. Note that nonlocal tubes that would end outside of the finite length *L* of the network are blocked.

Figure 1a shows a photograph of one of the considered 1D nonlocal periodic fluid‐flow networks. The network has been assembled by standard microfluidic tubes (transparent PVC tubing, Druckluft‐Fachhandel, Germany) and connectors (Push‐in T‐connector: NPQE‐T‐Q6‐E‐F1A‐P10, Push‐in X‐connector: QSMX‐6, Ball valve: QH‐QS‐6, from FESTO company). All tubes are optically transparent and have an inner diameter of 2*r*  =  4 mm and an outer diameter of 6 mm. All local tubes have a length of *L*_1_ =  5 cm, all nonlocal tubes have a length of *L*_*N* ≥ 2_ =  28 cm. The networks are mounted onto a 3D printed holders (black) and all have a period or lattice constant of *a*  =  6 cm. The schematic below the photograph in Figure 1a is a discrete equivalent fluid‐flow circuit in which the connectors are represented as dots and the tubes as fluid‐flow resistors. Such networks can be seen as metamaterials when described as nonlocal continua by effective‐medium parameters, for example by an effective fluid‐flow resistance. Importantly, the networks contain nearest‐neighbor connections and connections to the *N*‐th nearest neighbor to the left and right, with *N*  =  2, *N*  =  3, and *N*  =  4 in Figure 1a, b, and c, respectively.

The design of these fluid‐flow networks has been inspired by analogy to our previous work on networks of electrical resistors [28] seen as metawires exhibiting nonlocal electrical conduction in the quasi‐static limit. To make the transition from Ohm's law of electrical conduction to Hagen‐Poiseuille's law of fluid flow, the electrical resistors need to be replaced by fluid‐flow resistors, voltages are replaced by pressure differences, and electrical (charge) currents by fluid (mass) currents. The lower part of Figure 1a illustrates this connection by showing a resistor network where the resistors can be interpreted either as fluid‐flow resistors or as electrical resistors.

Using this analogy, the relative strength of fluid flow through the nonlocal connections to that through the local connections is given by the ratio *R*_1_/*R_N_*, where *R*  = *R*_1_  is the fluid‐flow resistance of the local tubes and *R*  =  *R*_*N* ≥ 2_ is the fluid‐flow resistance of the nonlocal tubes. We calculate the important ratio *R*_1_/*R_N_* from Hagen‐Poiseuille's law and the known inner radii and lengths of the local and nonlocal tubes (see above), respectively. For a straight cylindrical rigid tube of length *l* and inner radius *r*, Hagen‐Poiseuille's law states that the fluid‐flow resistance *R* is given by *R*  =  8η*l*/(π*r* ^4^), where η is the fluid shear viscosity. To obtain pronounced nonlocal effects, we aim at large ratios *R*_1_/*R_N_* ≫ 1 (see below). With identical inner tube diameters 2*r* (see above), we would rather have *R*_1_/ *R_N_* = *L*_1_ /*L_N_* ≈ 0.18 < 1. We therefore increase the fluid‐flow resistance of all local tubes *R*_1_ by inserting a constriction with inner diameter *d*  =  2*r*  =  0.5 mm and length *l*  =  3 mm made from aluminum, which is squeezed into the tubes (see inset on the right‐hand side in Figure 1a). The flow resistance of the constriction is again calculated from Hagen‐Poiseuille's law. This resistance adds to the flow resistance of each remaining local tube. The resulting ratios *R*_1_/*R_N_* ≈ 42 ≫ 1 will be quoted below.

**FIGURE 2:**
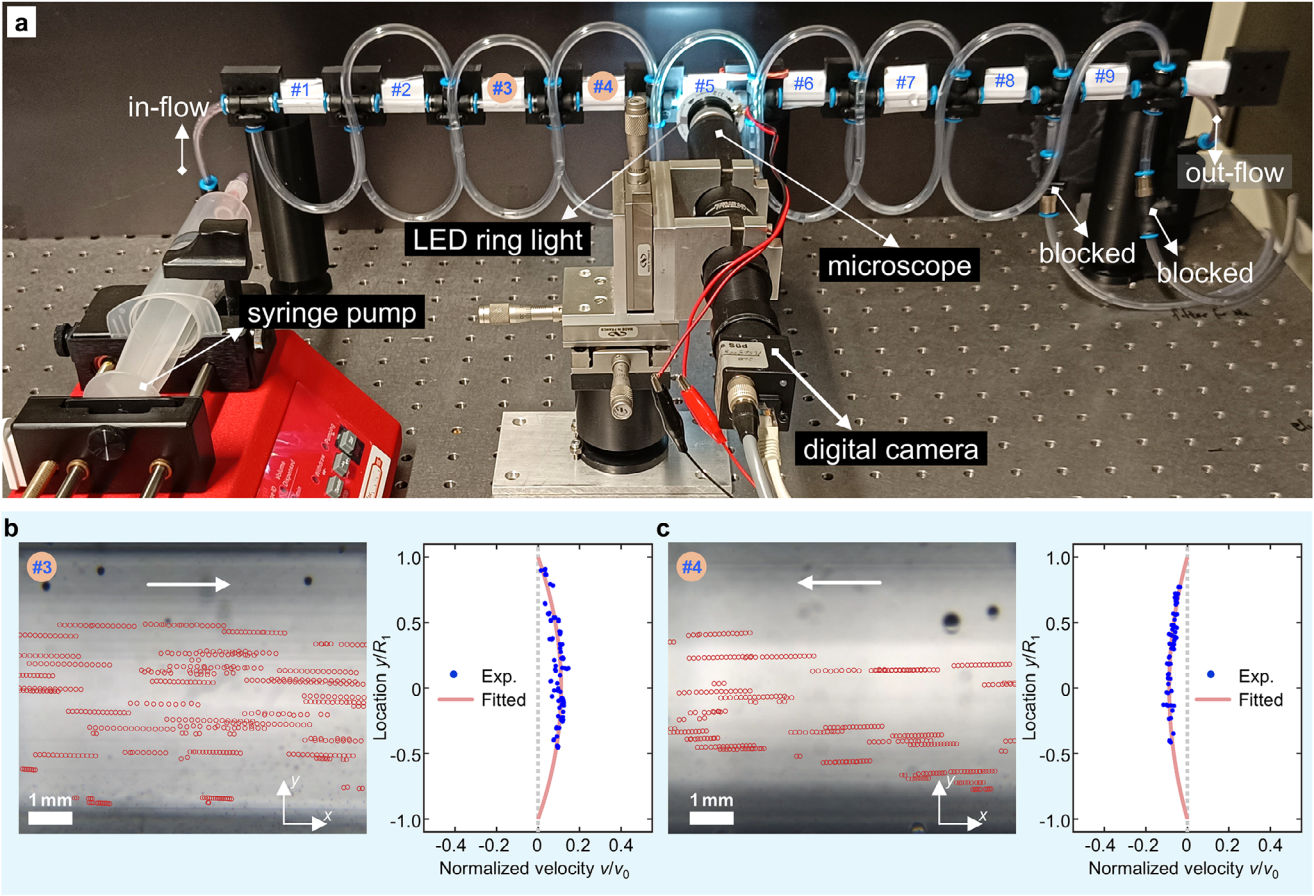
Tracking of fluid flow inside a tube‐network metamaterial. (a) Photograph of the experimental setup. Water is pumped into the tube network from the left via a syringe pump. Fluid flow inside the tubes is imaged from the side with a digital microscope. Spherical polystyrene particles with a diameter of 10 µm are used for tracking. The flow velocity is derived by tracking the polystyrene particles from recorded videos and using a particle‐tracking velocimetry algorithm. Examples for the local tubes #3 and #4 are given in (b) and (c), respectively (also refer to Movie S1). (b) Red circles indicate tracked positions of the polystyrene particles from all frames of the recorded video. The arrows indicate the derived flow direction. The panel on the right shows the distribution of the measured flow velocity (blue dots) along the transverse direction of the tube. We fit the experimental data with a parabola, with the amplitude, the center position, and the curvature as fitting parameters. The amplitude provides us with the measured velocity in the tube. (c) Same as (b), but the local tube at position #4 in (a). See Methods for more details on the particle‐tracking velocimetry.

The setup for the fluid‐flow experiments is shown in Figure 2a. Before starting the experiment, we fill the tube network with water, mixed with polystyrene microparticles (PS‐R‐10.0,10 µm diameter, microparticles GmbH, Germany). These microparticles have a mass density of 1.05 g cm^−3^, which is close to that of water. This minimizes sedimentation that would occur for a larger mass density of the microparticles. We exert caution that the tube network is fully filled with water to reduce the influence of air bubbles. During the experiment, a motorized syringe pump is used to continuously inject water into the micro‐fluidic network. The in‐flow velocity, *v*_in_, is adjusted to be sufficiently low to lead to a laminar fluid flow and to be sufficiently high to make the particle‐tracking algorithm (based on differences between subsequent images) work reliably. For different samples, the mean in‐flow velocity, *v*_in_, is chosen accordingly and has been in the range between *v*_in_ =  0.53 and 4.2 mm s − 1. The extracted velocities are normalized with respect to this in‐flow velocity. Under these conditions, the Reynolds number at the entrance tube is given by Re  = *v*_in_ 2*r*ρ/η with the fluid mass density ρ and the fluid shear viscosity η. For water we choose ρ  =  1000 kg m^−3^ and η  =  0.001 Pa s. With *r*  =  2 mm for the entrance tube (see above), we obtain Reynolds numbers in the range from 2.12 to 16.8. Note that the local Reynolds numbers within the tube network may be larger, especially in and near the constrictions within the local tubes. However, we have not observed any velocity‐dependent effects under these conditions.

In order to optically resolve the micrometer‐sized particles in the fluid flow, we have built a digital microscope with an infinite‐corrected objective lens (PL 3.2 × /0.06, effective focus length 75 mm, LEITZ WETZLAR, Germany) and a tube lens (AC254‐125‐A‐ML, focus length 125 mm, Thorlabs, USA). A ring‐type LED is mounted on the objective lens to provide illumination. White pieces of paper are placed behind the tubes to diffusively reflect light from the rear side to obtain high‐contrast images. We record the fluid flow in the tubes by using a digital camera (BFLY‐PGE‐50H5C‐C, Point Gray Research inc., Canada) at a frame rate of 7.5 Hz, 2448 pixels in width and 2048 pixels in height. Each pixel has a side length of about 2.16 µm. The microscope is adjusted such that the focal plane roughly lies in the middle of the tubes and that the flow direction is horizontal. For each measured position, we record a video with a duration of about 6 s, equivalent to 45 individual image frames. The details of the particle‐tracking analysis based on these videos are described in Methods.

**FIGURE 3:**
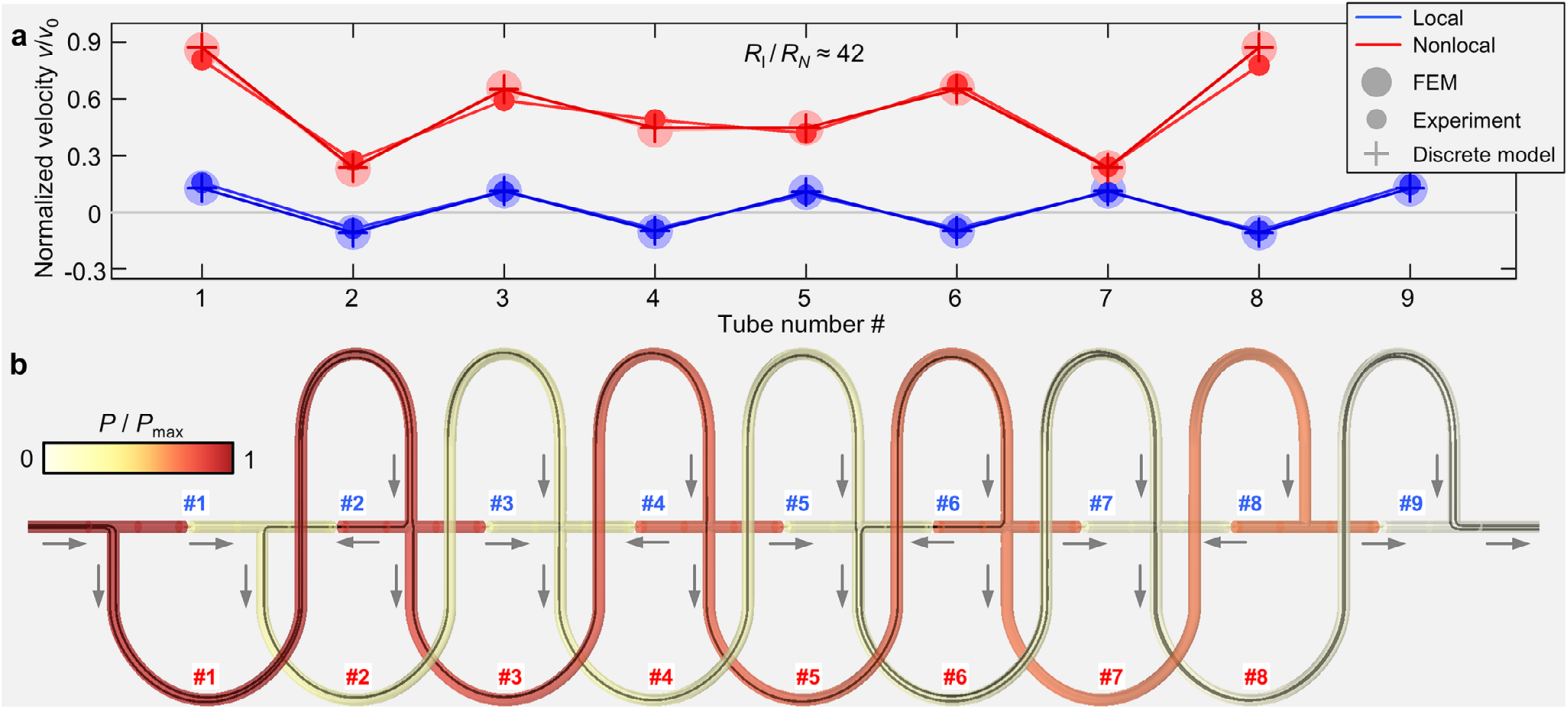
Fluid flow characteristic in nonlocal microfluidic metamaterial. (a) Flow velocity, normalized to the inflow velocity, in the local (blue) and nonlocal tubes (red) derived from the experiments (small dots), from finite‐element‐method (FEM) calculations (larger dots), and from a simple discrete model (crosses). The results from a systematic parameter variation are shown in Figure 4. (b) False‐color representation of the calculated pressure field under laminar‐flow conditions. Gray arrows indicate the fluid‐flow direction in the corresponding tubes. Note that flow in some local tubes is backwards, i.e., opposite to the main flow. The two black lines inside the tubes are example streamlines. A more complete set of streamlines (with constriction) is given in Figure 5, with the corresponding case without constriction shown in Figure S1.

Experimental results for *N*  =  2, *R*_1_/ *R_N_* =  42, and a metamaterial with a length of *L*  =  9*a*, are summarized in Figure 3a by the small dots. Blue color refers to the local tubes, red color to the nonlocal tubes. To allow for a direct comparison, results from finite‐element‐method (FEM) calculations are depicted in the same figure by larger dots, results obtained from a simple discrete model by crosses (also see figure legend). The two theory approaches are described in detail below. We find that experiment and theory comprise forward (positive velocity) and backward (negative velocity) flows. Figure 3b exhibits corresponding numerical FEM calculations of the pressure field throughout the entire tube network depicted on a false‐color scale. The pressure field anomalously oscillates with a period of *Na*  =  2*a*, leading to alternating forward/backward flows indicated schematically by the arrows in this panel.

**FIGURE 4:**
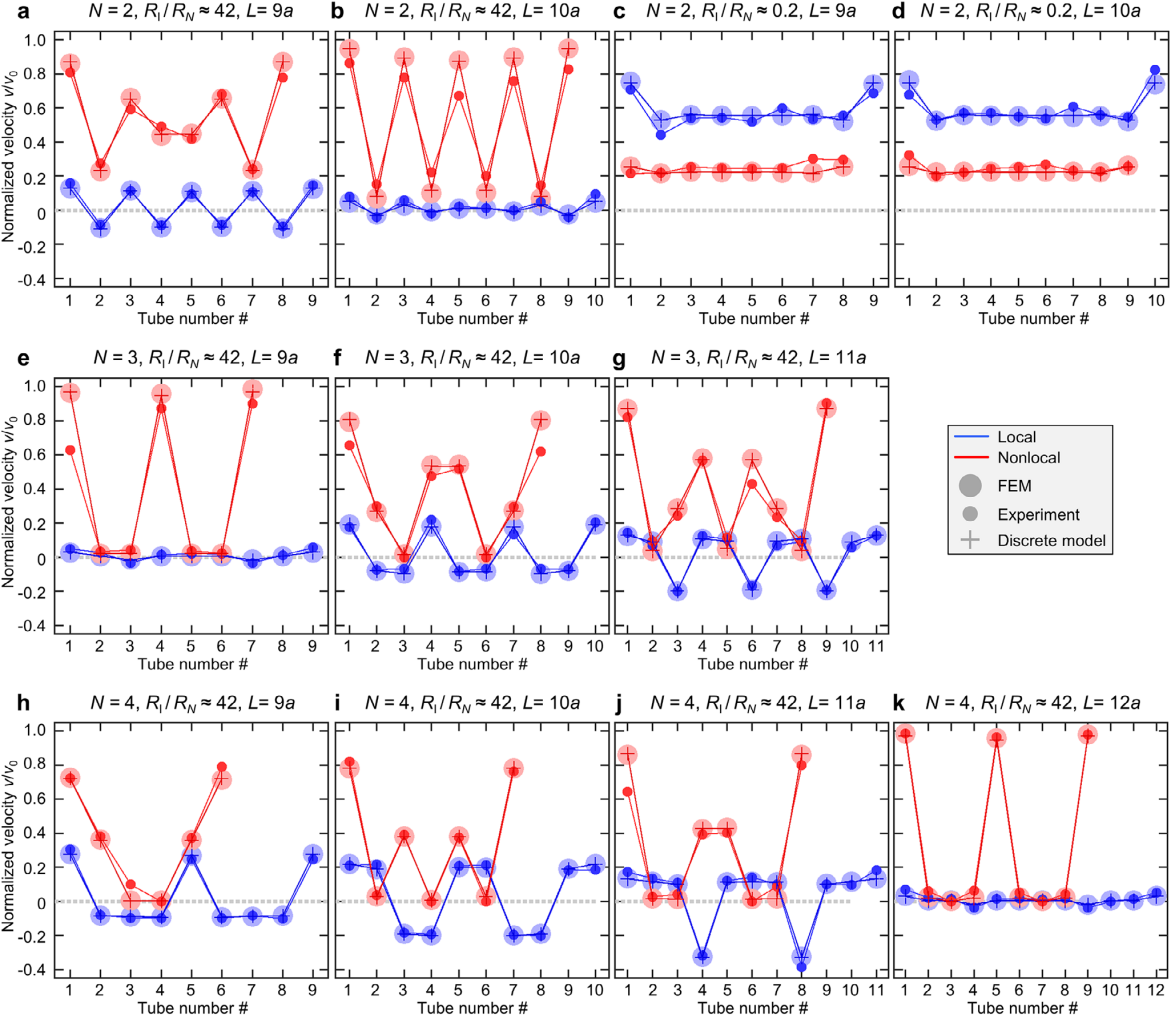
Dependence of flow profiles on nonlocal interaction order, connection‐strength ratio, and sample length. (a) Normalized flow velocity in each local tube (blue) and nonlocal tube (red) of the metamaterial with strong second‐order nonlocal interaction, *N*  =  2, *R*_1_/*R_N_* =  42, and length *L*  =  9*a*. FEM calculation, experiment measurements, and results from a discrete model calculation are represented by larger dots, small dots, and crosses, respectively (cf. Figure 3a). (b) Same as (a), but for a longer sample, *L*  =  10*a*. (c,d) Same as (a,b), but for weak nonlocal interaction, *R*_1_/*R_N_* =  0.2. The flow behavior is rather uniform in the local tubes and nonlocal tubes, apart from some edge effects at the metamaterial ends. (e–g) for the metamaterial with strong third‐order (*N*  =  3) nonlocal interaction *R*_1_/*R_N_* =  42. (h–k) For the metamaterial with strong fourth‐order (*N*  =  4) nonlocal interaction *R*_1_/ *R_N_* =  42. Oscillations of the flow velocity are clearly visible in both, the local and the nonlocal tubes for the case of strong nonlocal interactions, *R*_1_/ *R_N_* =  42. The oscillation period is *Na*. The fluid flow in the local tubes (blue symbols) oscillates between backwards (negative) and forward (positive), whereas it is always positive for the nonlocal tubes (red symbols).

The result of a systematic parameter variation is shown in Figure 4. To connect to Figure 3a, Figure 4a repeats results for the case of *N*  =  2 within the nonlocal regime (*R*_1_/ *R_N_* =  42) and for a length of the 1D microfluidic chain of *L*  =  9*a*. In Figure 4b, the parameters are the same, except for the length of *L*  =  10*a*. The corresponding controls within the local regime (*R*_1_/*R_N_* ≈ 0.2), shown in panels c and d of Figure 4 for otherwise identical parameters to panels a and b, exhibit no visible oscillations. Figure 4e–g show results within the nonlocal regime (*R*_1_/ *R_N_* =  42) for the case of *N*  =  3 for three different lengths *L*  =  9*a*,  10*a*,  11*a*. Likewise, Figure 4h–k shows results within the nonlocal regime (*R*_1_/ *R_N_* =  42) for the case of *N*  =  4 for three different lengths *L*  =  9*a*,  10*a*,  11*a*, 12*a*. For all three cases *N*  =  2,  3,  4 (i.e., the three rows of Figure 4), the amplitude of the spatial oscillations versus position changes as a result of different boundary conditions due to the different lengths, but the oscillation period and the fact that we do obtain alternating backward and forward flows is robust and only depends on the nonlocal order *N* for sufficiently strong nonlocality, i.e., for sufficiently large ratios *R*_1_/*R_N_*.

### 2.2 Theory

The experimental data points (small dots) in Figures 3 and 4 are in very good agreement with both, finite‐element‐method (FEM) calculations in the laminar‐flow regime and solutions of a simplified discrete model. Results for both have also already been depicted in Figure 3 as large dots and crosses, respectively. In what follows, we describe both of these approaches. We start with the discussion of the discrete model, aiming at providing the reader with an intuitive understanding via the zero‐frequency evanescent Bloch eigenmodes of the periodic fluid‐flow network. Next, we discuss additional numerical results on the fluid‐flow streamlines, especially under conditions where backward fluid‐flows occur. We show that the backward flows are connected with loop‐like behavior of the streamlines.

For the stationary flow of an incompressible liquid, we can start from the continuity equation and apply that to the *n*‐th connector (n=1,…,L/a) of the fluid‐flow network (compare Figure 1). Considering an infinitely periodic 1D network, each connector has two inflows and two outflows via the tubes. In the language of graph theory, the connectors are ‘points‘ (or ‘vertices‘ or ‘nodes‘) and the tubes are the ‘connections‘ (or ‘edges‘ or ‘links‘). The ‘degree’ is 2 + 2  =  4. Together with Hagen‐Poiseuille's law, we obtain

(1) $$\left(\frac{P_{n}-P_{n-1}}{R_{1}}+\frac{P_{n}-P_{n-N}}{R_{N}}\right)-\;\left(\frac{P_{n+1}-P_{n}}{R_{1}}+\frac{P_{n+N}-P_{n}}{R_{N}}\right)=0$$

In analogy to our previous work on electrical conduction [28] and static elasticity [24], we make the ansatz for the static Bloch eigenfunctions

(2) $$P_{n}=\tilde{P}\;\exp\left(i\left(kan-\omega t\right)\right)$$

with prefactor $\tilde{P}$, wavenumber or spatial frequency *k*, and frequency ω  =  0. Inserting this ansatz into Equation (1), we obtain the implicit equation for *k* given by

(3) $$0=\left(1-\cos\left(ka\right)\right)+\frac{R_{1}}{R_{N}}\;\left(1-\cos\left(Nka\right)\right)$$

We find 2(*N* − 1) complex‐valued solutions k1,…,2(N−1)=Re(k)+iIm(k) that depend on the dimensionless ratio *R*_1_/*R_N_*. The real part of *k* determines the spatial oscillation period or wavelength λ of the pressure field via λ  =  2π/|Re(*k*)|. The inverse of the imaginary part, *l*  =  1/|Im(*k*)|, is the exponential decay length of this oscillation. For example, for *R*_1_/ *R_N_* =  42 and *N*  =  2,  3,  4, we obtain λ ≈ 2*a*,  3*a*,  4*a* and *l* ≈ 6.5*a*,  11.3*a*,  18.4*a* by numerical solution. For *R*_1_/ *R_N_* =  0.18 and *N*  =  2,  3,  4, we obtain λ ≈ 2*a*,  3*a*,  4*a* and *l* ≈ 0.50*a*,  0.96*a*,  1.20*a*. This means that there is no threshold for the ratio *R*_1_/*R_N_* in Equation (3), which has complex‐valued solutions for *k* for any finite ratio $R_{1}/R_{N}\neq 0$. From Equation (3), we derive that the decay length asymptotically tends to zero according to $l=-1/\mathrm{In}(R_{1}/R_{N})\to 0$ for $R_{1}/R_{N}\to 0$. In Figure 4c,d, where *R*_1_/ *R_N_* =  0.18, no apparent oscillations are visible.

Intuitively, for example for *N*  =  2 and *R*_1_/*R_N_* ≫ 1,  the pressure field oscillates with period 2*a*. Hence the pressure difference between adjacent connectors or sites, *P_n_* − *P*_*n* − 1_, oscillates with alternating sign. Together with Hagen‐Poiseuille's law, this means that the fluid‐flow current oscillates between positive (forward) and negative (backward) – in agreement with the above experiments. For *N*  =  2 and *R*_1_/ *R_N_* =  0.18, no visible backward currents appear – in agreement with experiment. The behavior for *N* ≥ 3 is analogous, except for a larger period of λ ≈ 3*a* and λ ≈ 4*a*, respectively.

For a network with finite length *L* instead of an infinitely periodic network, the solutions are a linear combination of all eigenfunctions with prefactors chosen to match the boundary conditions [24]. At this point, the boundary conditions enter explicitly—in agreement with the above experiments. A detailed comparison between experiment (small dots) and discrete model (crosses) is given in Figures 3a and 4. As the eigenfunctions enter into all observables via the boundary conditions (e.g., different numbers of unit cells in the network), the fluid current for fixed pressure difference between the left and the right end of the network also oscillates as a function of the integer ratio of network length to period, *L*/*a* (not depicted). Counter‐intuitively, this includes the possibility that the fluid current increases when increasing the ratio *L*/*a*. Mathematically, due to the analogy between Hagen‐Poiseuille's law and Ohm's law, this behavior is identical to that previously discussed for electrical metamaterials in ref. [28].

Next, we numerically calculate the fluid‐flow in the tube‐network metamaterials by using the ‘Laminar Flow Module’ in the commercial software COMSOL Multiphysics. The pressure field, *P*(**r**), and the velocity field, **v**(**r**), are obtained from solving the steady‐state incompressible Navier‐Stokes equations in three dimensions

(4) $$\rho \left(v\left(r\right)\cdot \nabla \right)v\left(r\right)=\nabla \cdot \left[-PI+\eta \left(\nabla \left(v\left(r\right)\right)+\left(v\left(r\right)\right)\nabla \right)\right]$$

combined with the continuity equation

(5) $$\rho \nabla \cdot v\;\left(r\right)=0$$

ρ denotes the fluid mass density, **I** the identity tensor, and η is the fluid shear viscosity. For water at room temperature, we again (see above) choose ρ  =  1000 kg m^−3^ and η  =  0.001 Pa s. We impose an inlet flow boundary at the left side of the metamaterial model (see Figure 3b), and an outlet boundary with pressure *P*  =  0 at the right end of the model. All other walls of the metamaterials are assumed to be non‐slippery boundary conditions (**v**  =  0).

**FIGURE 5:**
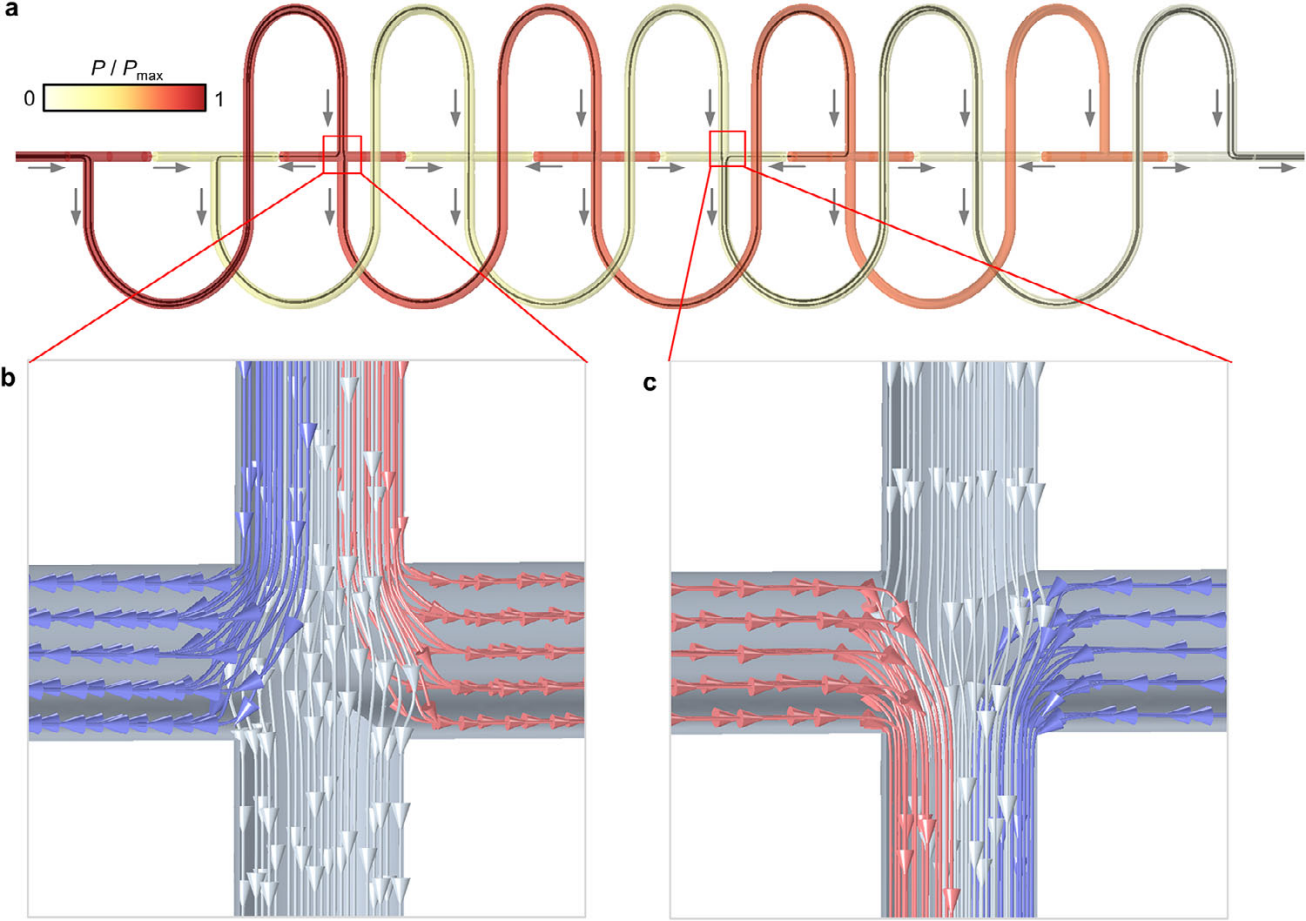
Calculated streamlines of fluid flow. (a) Same as Figure 3b, but with two red boxes indicating the regions magnified in panels (b) and (c), respectively. (b) and (c) Fluid‐flow behavior at the two four‐way connectors indicated in (a), at which two local tubes (horizontal direction) and two nonlocal tubes (vertical direction) meet. Arrows represent the local direction of fluid flow. The colors red and blue further emphasize the positive (forward) or negative (backward) flow direction along the horizontal axis, respectively.

The combination of forward and backward flows leads to a complex behavior of the streamlines of fluid flow. Clearly, streamlines cannot cross. Therefore, laminar flow vortices in a strict sense are not possible. Yet, in many regions, forward and backward streamlines must coexist in the tubes. Only selected streamlines and flow directions have been shown in Figure 3b. Figure 5 illustrates how backward and forward streamlines split and merge, respectively, at a connector for two example connectors. Figure S1 shows the corresponding control results without the constrictions in the local tubes, i.e., for the limit of a local metamaterial in which no backward flows are visible.

Many streamlines are shown in Figure S2, where we group them according to which local tube (containing a constriction) they pass through. Clearly, the alternating backward/forward flows are connected to loop‐shaped streamlines of the water flow. While any one streamline has zero loops or only a single loop, water molecules may still undergo multiple loops/detours. Combined with diffusion and deviations from strictly laminar flow, this means that the discussed backward flows might potentially be interesting for improved mixing of different fluids. However, we have not performed experiments in this direction.

Finally, by means of FEM calculations, we study the robustness of the observed overall behavior against two aspects: higher Reynolds numbers and disorder. Corresponding results are shown in Figures S3 and S4, respectively. In Figure S3, we start from the parameters in Figure 4a (*L*  =  9*a*) and 4b (*L*  =  10*a*), respectively, and increase the Reynolds number at the input tube from Re  =  4.2 to 42 (i.e., 5 times) and 210 (i.e., 50 times). We find only minor quantitative changes except for the case in Figure S3c in which the relative weight of the local flows has increased with respect to Figure 4a. However, the oscillatory behavior stays robust. For yet larger Reynolds numbers at the entrance tube by about a factor of two (not depicted) relative to the already 50‐times‐increased case, the FEM calculations do not converge, indicating the onset of turbulent flow in the network, especially near the constrictions, where the local Reynolds numbers are considerably larger than in the input tube.

In Figure S4, we again start from the parameters in Figure 4a (*N*  =  2) and introduce random disorder in the strength of the nonlocal couplings by locally varying the radius of the flow constrictions in the local tubes. As their resistance is inversely proportional to the fourth power of their inner radius *r_n_*, and as this flow resistance dominates the total resistance of the local tubes, this variation introduces a strong perturbation. We choose the inner radius of the constriction *r_n_* in local tube #*n* according to $r=\mathrm{const}.=0.5\;\mathrm{mm}\;=r_{n}\to r_{n}\left(1+0.5\times p_{n}\right)$, where the *p_n_* are random numbers evenly distributed in the interval [− 1, +1]. This means that the inner radius can change by as much as a factor of three, and hence the flow resistance of the constriction by a factor of 3^4^ =  81. In Figure S4, we repeat the results from Figure 4a (no disorder) together with three different disorder realizations (cases 1–3). We find quantitative changes of the water‐flow velocities in the different local and nonlocal tubes, but the oscillatory behavior connected to backward flows remains.

Altogether, we conclude that the observed behavior is robust against rather large disorder and higher Reynolds numbers, where water flow is no longer linear, but not yet turbulent.

## 3 Conclusion

The interplay of fluid flows and metamaterials has recently attracted attention [38] as an emerging field in which metamaterial design is driven by a number of specific applications. So far, this design process has been based on interactions with walls/surfaces, including the possibility of exploiting Bragg reflections in periodic structures as well as localized resonances [42, 43]. In the time domain, the frequency‐dependent response of a resonance corresponds to a behavior that is nonlocal in time, i.e., the causal behavior of the system at time *t* depends on the state of the system at many previous times *t*′ < *t*. Here, we have explored the possibility of a stationary (time‐independent) fluid‐flow behavior that is nonlocal in space, i.e., the causal behavior of the system at position *x* depends on the fluid flow at many other positions $x^{\prime }\neq x$. Such nonlocal behavior has previously been explored in many different systems [11, 12, 13], but not for fluid flows. Specifically, herein we have studied laminar water flow in one‐dimensionally periodic networks of tubes and connectors. These networks comprise nearest‐neighbor (local) and beyond‐nearest‐neighbor (nonlocal) connections of different orders *N*  =  2,  3,  4. For sufficiently strong nonlocal connections, we find spatially alternating backward/forward water flows. The backward flows are reminiscent of backward waves (= negative refractive indices) in wave problems [44, 45] with which the entire field of metamaterials started about a quarter of a century ago [30, 46, 47]. More broadly speaking, nonlocal interactions add one more design tool for engineering fluid flows by metamaterials [38]. We speculate that the backwards flows discussed in this paper might potentially be interesting for improved mixing of different fluids. However, such mixing requires deviations from laminar flow and fluid diffusion in addition to fluid flow.

## 4 Methods

The fluid‐flow velocities are extracted by analyzing the recorded videos of the microparticle motion. Each recorded video contains three channels (RGB channels). We use the green (G) channel, which has turned out to provide the best image contrast. The particle‐tracking velocimetry algorithm is composed of three main steps: The first step is to determine all particles and their positions in each frame of the recorded video. For each frame, we use the difference between one frame and the next one. The last frame is used as a buffer frame. This operation filters out stationary features, such as particles or dirt stuck on the inner walls of the tube, and only leaves moving particles. The data are then binarized according to a threshold value. Pixels corresponding to moving particles appear as white, motionless background becomes black. A threshold value of 5 gives good tracking quality and has been chosen for all videos. From the binarized data, we find clusters of pixels that are connected together by using the Matlab built‐in function ‘bwconncomp‘, which requires ‘Image Processing Toolbox‘. Only pixel clusters with more than 25 pixels (with a diameter of about 6 µm) are registered as particles. The center positions of these pixel clusters are identified as the positions of particles in current frame. The second step is to assign particles to correct trajectories [48]. We start from the second frame. For each particle position, **r**_current_, in current frame, we find its closest particle position, **r**_previous_, from all particle positions in the previous frame. Additionally, we double check whether the position **r**_current_ among all particle in current frame is the closest to position **r**_previous_. If so, this is a correct assignment, otherwise this association is abandoned. Likewise, for particle position **r**_current_ in a current frame, we find its closest particle position, **r**_next_, from all particle positions in next frame. Again, this matching is double‐checked to ensure correct association. We note that the double‐checking procedure makes the algorithm robust against certain edge cases, such as crossing particle trajectories or ‘disappearing’ particles due to out‐of‐focus movement [48]. The three identified positions in three consecutive frames form a valid short trajectory $r_{\mathrm{previous}}\to r_{\mathrm{current}}\to r_{\mathrm{next}}$. If the particle positions **r**_previous_ and **r**_current_ are already assigned to a trajectory, then position **r**_next_ is added to the trajectory, otherwise, a new trajectory is assigned. The above algorithm allows us to identify particle trajectories from the recorded videos. See Movie S1 for two example videos with tracked particles marked by circles. In the third step, we determine the maximum velocity of the fluid flow. For each registered trajectory in the previous step, we derive its average velocity and its average vertical position (see Figure 2b). For laminar water flow in the tubes, the flow velocity is expected to exhibit a parabolic profile, with the velocity being zero at the tube inner walls and being maximal in the center of the tube. Therefore, by using a least‐square algorithm we fit a parabolic function to the experimental velocity distribution, and derive the maximum velocity, *v*_max_, the center position, and the curvature. For each measured tube, the obtained maximum velocity is normalized to the in‐flow velocity, *v*_in_.

## Author Contributions

K.W., Y.C., and M.W. conceived the study. K.W. and Y.C. conducted the experiments and evaluated the theory. M.W. drafted the paper. All authors contributed to the interpretation of the data and the discussions. M.W. supervised the overall project.

## Funding

We acknowledge funding by the Deutsche Forschungsgemeinschaft (DFG, German Research Foundation) under Germany's Excellence Strategy for the Excellence Cluster ‘3D Matter Made to Order’ (2082/1–390761711; 2082/2–390761711), by the Carl Zeiss Foundation, and by the Helmholtz program Materials Systems Engineering.

## Conflicts of Interest

The authors declare no conflicts of interest.

## Supporting information




**Supporting File 1**: advs77912‐sup‐0001‐SuppMat.docx.

**Supporting File 2**: advs77912‐sup‐0002‐MovieS1.mp4.

## Data Availability

The data that support the plots within this paper and other findings of this study (including the COMSOL files) are published on the open access data repository of the Karlsruhe Institute of Technology (KIT) [https://doi.org/10.35097/j8gu1un4wg192241 ] and are additionally available from the corresponding authors upon reasonable request.
