# Supplementary material for: Experiments on Nonlocal Fluid‐Flow Metamaterials
Source: Adv Sci (Weinh). 2026 Sep 27:e77912. Online ahead of print. doi: 10.1002/advs.77912 (PMC13616373; doi:10.1002/advs.77912)
Supplement: Supplementary file 1 [file ADVS-9999-e77912-s002.docx]

Supporting Information

Experiments on nonlocal fluid-flow metamaterials

Ke Wang^*^, Yi Chen^*^, and Martin Wegener^*^

K. Wang, M. Wegener

Institute of Nanotechnology, Karlsruhe Institute of Technology (KIT), Karlsruhe 76131, Germany.

K. Wang, Y. Chen, M. Wegener

Institute of Applied Physics, Karlsruhe Institute of Technology (KIT), Karlsruhe 76131, Germany.

Y. Chen

Marine Science and Technology Domain, Beijing Institute of Technology (BIT), Zhuhai 519088, China

E-mail: [ke.wang2@partner.kit.edu](mailto:ke.wang2@partner.kit.edu) (K.W.); [yi.chen@partner.kit.edu](mailto:yi.chen@partner.kit.edu) (Y.C.); [martin.wegener@kit.edu](mailto:martin.wegener@kit.edu) (M.W.)

**Figure S1. FEM calculated fluid-flow streamlines.** Same as Figure 5, but without aluminum hollow cylinders (constrictions) inserted into the local tubes, i.e., for the regime of weak nonlocal interactions or $R_{1}/ R_{N}=0.18$. Here, the fluid flow is forward (positive) in all local and nonlocal tubes.

**Figure S2. FEM calculated laminar-flow streamlines inside the nonlocal microfluidic metamaterial in Figure 1a.** (a) – (e) show streamlines that pass through the first, second, third, fourth, and fifth local tube etc., respectively. All streamlines belong to the same setup, but they are separated and colored here for clarity. Each streamline must go through a local tube once, either in the backward or the forward direction. The metamaterial parameters are $N=2$, $R_{1}/R_{N}=42$, and $L/a=9$. Note that not all streamlines are shown for the sake of clarity (see “…” at the bottom).

**Figure S3. Normalized velocities for different Reynolds numbers.** (a)**-**(c) Normalized fluid flow velocities in the nearest- (blue) and second-nearest-neighbor (red) tubes for $N=2, R_{1}/R_{N}\approx42,$and $L=9a$, corresponding to Reynolds numbers at the input tube of $\mathrm{Re}=4.2, 42$, and 210, respectively. (d)**-**(f) Same as in (a)**-**(c), but for $L=10a$.

**Figure S4. Robustness of normalized velocity against random disorder in the nonlocal coupling strength.** (a) Same as FEM results in Figure 4a in the main text. (b)-(d) Normalized velocity with different random numbers $p_{n}$ ($n=1,2,\ldots9$), for specific random-number selections as summarized in **Table 1**.

**Table 1.** (Selection of the random numbers $p_{n}$ in three different cases)

| $n$ | 1 | 2 | 3 | 4 | 5 | 6 | 7 | 8 | 9 |
| --- | --- | --- | --- | --- | --- | --- | --- | --- | --- |
| Case 1 | 0.6294 | 0.8116 | -0.7460 | 0.8268 | 0.2647 | -0.8049 | -0.4430 | 0.0938 | 0.9150 |
| Case 2 | 0.9298 | -0.6848 | 0.9412 | 0.9143 | -0.0292 | 0.6006 | -0.7162 | -0.1565 | 0.8315 |
| Case 3 | 0.5844 | 0.9190 | 0.3115 | -0.9286 | 0.6983 | 0.8680 | 0.3575 | 0.5155 | 0.4863 |
